# Unfavorable Outcomes Related to Endovascular Treatment of Giant Vertebrobasilar Aneurysms

**DOI:** 10.3389/fneur.2020.00748

**Published:** 2020-08-11

**Authors:** Miao Li, Huaxin Liang, Jie Wang

**Affiliations:** ^1^Department of Neurosurgery, The China-Japan Union Hospital of Jilin University, Changchun, China; ^2^Department of Neurology, The China-Japan Union Hospital of Jilin University, Changchun, China

**Keywords:** giant vertebrobasilar aneurysms, endovascular treatment, outcome, complications, poor prognosis

## Abstract

**Background:** Giant vertebrobasilar aneurysms (GVBAs) have an unfavorable natural history if left untreated and often pose a sizeable challenge to endovascular treatment. The aim of this study was to analyze the angiographic and clinical outcomes of GVBAs treated by various endovascular procedures.

**Methods:** Between January 2010 and September 2018, 27 patients with 27 GVBAs treated endovascularly were enrolled in this consecutive study. The clinical and angiographic features, treatment modalities, and outcomes were analyzed.

**Results:** The patient cohort comprised 21 men (77.8%) and 6 women (22.2%) of mean age 42.7 ± 18.9 years (range, 6–65 years). The most common presenting symptom was compressive symptoms, present in 15 patients (55.6%). None of the GVBAs was ruptured. Of the 27 GVBAs, 23 aneurysms were dissecting aneurysm with intramural hematoma and 4 aneurysms were saccular. Regarding treatment approach, internal trapping was used in 5 aneurysms, stent-assisted coil embolization in 10, sole stenting in 4, and flow diverters in 8. Overall, 12 patients (44.4%) had an unfavorable angiographic or clinical outcome: 3 patients presented with post-operative complications and subsequent death, and 9 with poor prognosis during follow-up.

**Conclusions:** Patients with GVBAs may not benefit from endovascular treatment. Newer-generation devices are necessary to provide more optimal therapy for the management of these complex lesions.

## Introduction

Giant vertebrobasilar aneurysms (GVBAs), intracranial aneurysms with a maximum diameter of at least 25 mm originating from the vertebral and basilar artery, are rare and always challenging because of their complex neuroanatomy and pathophysiologic features (1, 2). Owing to minimal invasiveness and lower risk, endovascular treatment of GVBAs is considered to be safer than open surgery (3, 4). Although previous studies showed that endovascular treatment of GVBAs was always associated with high recurrence rates (5–7), these studies did not include cases with implantation of a flow diverter. Therefore, the aim of this study was to analyze the angiographic and clinical outcome of GVBAs treated endovascularly with stent-assisted coiling, overlapping stents, internal trapping, or flow diverters. The findings of this study should expand the knowledge base regarding preferences in clinical practice.

## Materials and Methods

### Patient Selection

This retrospective study was approved by our institutional ethics committee. Written informed consent was provided by patients or their relatives during hospitalization, and the privacy of the patients was strictly protected. Between January 2010 and September 2018, a total of 27 patients with 27 GVBAs treated endovascularly were enrolled in this study. The exclusion criteria included the following: (1) pre-existing diagnoses of arteritis, fibromuscular dysplasia, iatrogenic aneurysms, or pseudoaneurysms; (2) history of traumatic and iatrogenic injury; (3) extracranial dissecting aneurysms extending into the intracranial segment; (4) GVBAs without endovascular treatment; (5) aneurysm size <25 mm; (6) vertebrobasilar dolichoectasia. The information collected and analyzed included patient demographics (age and sex), location and angiographic features of the GVBAs, endovascular treatment selected, treatment complications, follow-up interval, and angiographic and clinical follow-up outcomes.

### Endovascular Procedures

**Figure 1:**
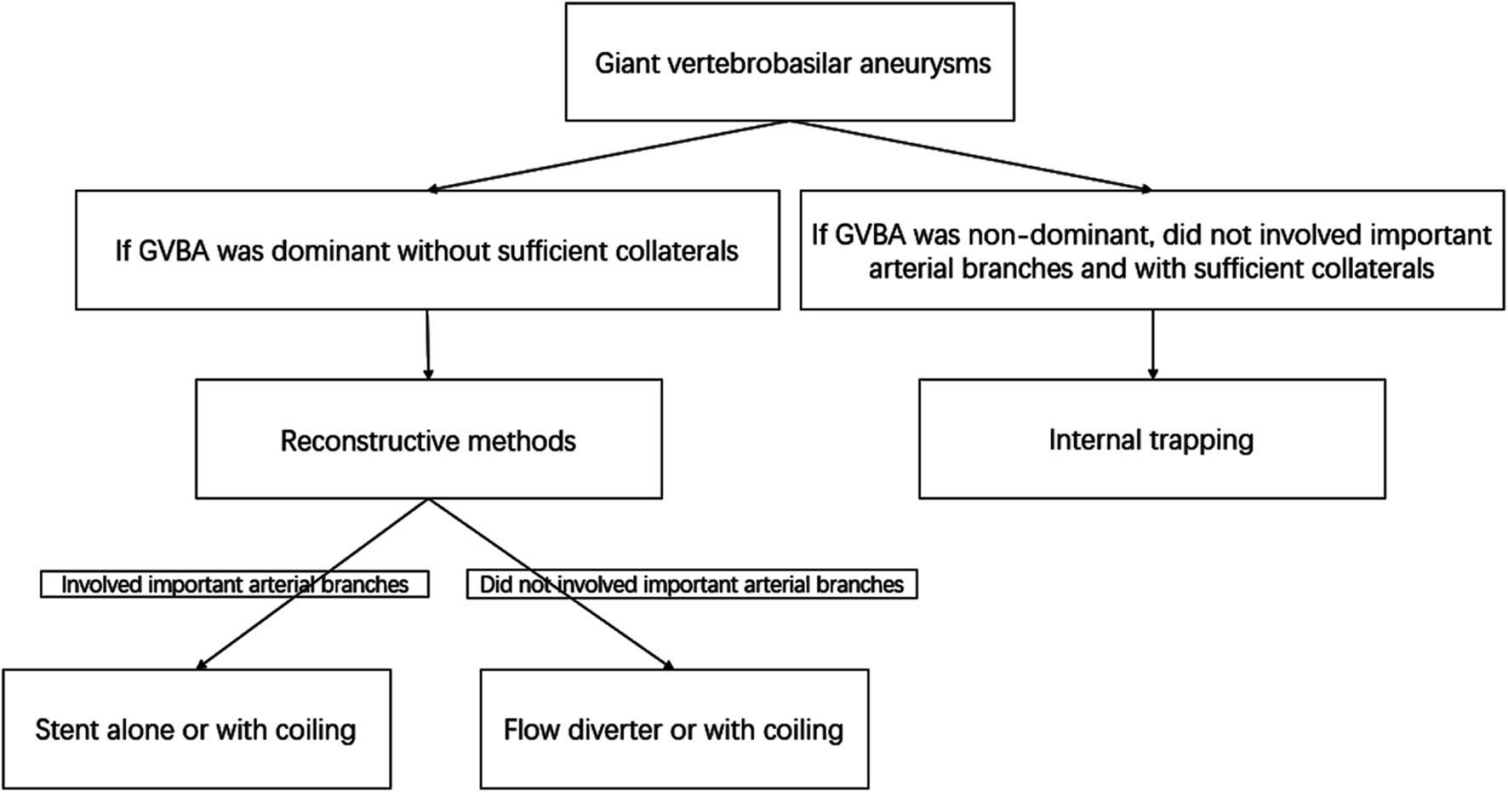
Flow chart of the decision concerning endovascular treatment methods for giant vertebrobasilar aneurysms.

Endovascular treatment was performed under general anesthesia and systemic intravenous heparin. Patients were treated with internal trapping, overlapping stents, stent-assisted coiling, or flow diverters as appropriate. For internal trapping, various platinum coils were used to occlude the dissecting aneurysm and the parent artery. Balloon occlusion test was used to determine whether sufficient collateral circulation compensate after the vessel sacrificed. Internal trapping was our first choice if the GVBAs did not involve the dominant vertebral artery or the important arterial branches (such as posterior inferior cerebellar artery, anterior inferior cerebellar artery, or other large perforating arteries), and the collateral blood supply were confirmed to be away from the section of the blood vessel harboring the aneurysm. In contrast, if a GVBA was dominant without sufficient collaterals, the GVBA was treated with reconstructive methods using stents alone and flow diverter or with coiling. However, flow diverter was the first choice if a GVBA did not involve important arterial branches (Figure 1). If reconstructive endovascular procedures were chosen, patients were premedicated with a dual-antiplatelet regimen (75 mg of clopidogrel and 100 mg of aspirin daily) at least 5 days before the procedure. After the procedure, patients treated with a conventional stent were given 75 mg/d clopidogrel for 6 weeks and 100 mg/d aspirin for 6 months, while patients treated with a flow diverter were given 75 mg/d clopidogrel for 3 months and 100 mg/d aspirin thereafter.

### Materials

Various types of embolic materials used in the endovascular treatment included detachable coils such as the Matrix coil (Cordis, New Brunswick, NJ, USA) and Microplex coil (MicroVention, Aliso Viejo, CA, USA). Neurovascular stents were used to reconstruct the dissected artery, such as Enterprise (Cordis Neurovascular, Miami, FL, USA), Solitaire AB (Ev3, Irvine, CA, USA), and Low-profile Visualized Intraluminal Support (MicroVention Terumo, Tustin, CA, USA) stents, a silk flow-diverter stent (Balt Extrusion, Montmorency, France), and a pipeline embolization device (Covidien/ev3 Neurovascular, Irvine, CA, USA).

### Follow-Up

All patients were recommended to undergo a 6-month angiographic follow-up, and a magnetic resonance (MR) angiogram or computed tomography angiogram performed annually thereafter. Any aneurysm that displayed an increasing percentage of contrast filling of the aneurysmal sac on follow-up angiography or presented with more than 1-mm enlargement on MR imaging was considered an unfavorable angiographic outcome (8). The occlusion rate (<90%) for the aneurysms with flow diverter and re-patency of parent artery for the aneurysms with internal trapping were defined as unfavorable angiographic outcomes (9). Otherwise, the aneurysm was regarded as a favorable angiographic outcome. Patients' clinical outcomes were measured by the Glasgow Outcome Scale score at follow-up visits or by a telephone interview. The GOS score of 5 or 4 was considered a favorable clinical outcome, and scores of 3, 2, or 1 was unfavorable (10).

## Results

Between January 2010 and September 2018, 27 patients with 27 GVBAs were enrolled in the study. The clinical characteristics, imaging features, endovascular treatment therapies, and follow-up outcomes are listed in Table 1. Among the 27 patients, 12 (44.4%) had an unfavorable angiographic or clinical outcome. Three of these patients presented with post-operative complications and subsequent death while 9 carried a poor prognosis during follow-up.

**Table 1:** Details of 27 patients with giant vertebrobasilar aneurysms.

**Case No**.	**Gender**	**Age**	**Location**	**Size (mm)**	**Aneurysm type**	**Initial symptom**	**Endovascular treatment**	**Stents**	**Angiographic follow-up result**	**Angiographic follow-up time(m)**	**Clinical follow-up result (GOS)**	**Clinical follow-up time(m)**
1	M	54	VA	34.1	Dissecting	Compressive symptom	Stent-assisted coiling	Enterprise × 3	Unfavorable	4	4	24
2	M	47	VA	39.4	Dissecting	Compressive symptom	Stent-assisted coiling	Solitaire × 1	N/A	N/A	1	10
3	M	65	VA	35.2	Dissecting	Compessive symptom	Stent-assisted coiling	Enterprise × 1 + Solitaire × 1	Favorable	6	4	12
4	M	58	VA	27.7	Dissecting	Compessive symptom	Sole stenting	Enterprise × 1 + Solitaire × 1	Unfavorable	24	4	30
5	M	49	VA	26.9	Dissecting	Compessive symptom	Stent-assisted coiling	Enterprise × 1 + Solitaire × 1	Favorable	6	4	30
6	M	48	VA	25.2	Dissecting	Headache	Sole stenting	Enterprise × 3	Unfavorable	6	4	12
7	M	54	VA	26.2	Dissecting	Ischemic stroke	Stent-assisted coiling	Enterprise × 1 + Solitaire × 1	Favorable	6	4	6
8	M	62	VA	25.7	Dissecting	Headache	Stent-assisted coiling	Enterprise × 2 + LVIS × 1	Favorable	4	5	10
9	M	51	VA	36.5	Dissecting	Compressive symptom	Stent-assisted coiling	Enterprise × 2	Unfavorable	6	1	12
10	M	61	VA	25.4	Dissecting	Compressive symptom	Internal trapping	N/A	Favorable	6	4	12
11	M	52	VA	38.3	Dissecting	Compressive symptom	Internal trapping	N/A	Favorable	7	4	10
12	M	49	VA	32.5	Dissecting	Compressive symptom	Stent-assisted coiling	Enterprise × 2	Favorable	5	4	12
13	M	47	VA	25	Dissecting	Headache	Sole stenting	Enterprise × 2	Unfavorable	12	4	32
14	F	34	VA	25.9	Dissecting	Headache	Sole stenting	LVIS × 1	Favorable	6	5	12
15	M	58	VA	30.4	Dissecting	Headache	Stent-assisted coiling	Enterprise × 3	Unfavorable	6	1	14
16	M	52	VA	26.2	Dissecting	Incidental	Stent-assisted coiling	Enterprise × 2	Favorable	6	5	15
17	M	54	VA	28.3	Saccular	Headache	Flow diverter with adjunctive coiling	PED × 1	Favorable	8	5	12
18	F	55	VBJ	25.2	Saccular	Compressive symptom	Flow diverter with adjunctive coiling + contralateral VA sacrifice	PED × 1	N/A	N/A	1	N/A
19	M	12	BA	25.3	Saccular	Headache	Flow diverters	PED × 4	N/A	N/A	1	N/A
20	M	11	BA	30.3	Dissecting	Headache	Internal trapping	N/A	favorable	5	5	18
21	F	48	VA	26.4	Dissecting	Ischemic stroke	Flow diverter	PED × 1	Favorable	7	5	12
22	F	6	VA	28.9	Dissecting	Compressive symptom	Internal trapping	Internal trapping	Unfavorable	6	4	10
23	M	57	VA	25.8	Dissecting	Compressive symptom	Flow diverter	PED × 1	Favorable	5	4	11
24	M	37	VBJ	30.4	Saccular	Compressive symptom	Flow diverter with adjunctive coiling + contralateral VA sacrifice	Silk × 1	N/A	N/A	1	N/A
25	F	12	BA	34.2	Dissecting	Compressive symptom	Internal trapping	Internal trapping	Unfavorable	5	3	6
26	F	11	VA	25.6	Dissecting	Headache	Flow diverter with adjunctive coiling	PED × 1	Favorable	7	5	15
27	M	10	VBJ	28.3	Dissecting	Compressive symptom	Flow diverter with adjunctive coiling + contralateral VA sacrifice	PED × 1	Favorable	6	4	12

*M, male; F, female; VA, vertebral artery; BA, basilar artery; VBJ, vertebrobasilar junction; N/A, not applicable; GOS, Glasgow Outcome Scale score*.

### Clinical and Imaging Characteristics

**Figure 2:**
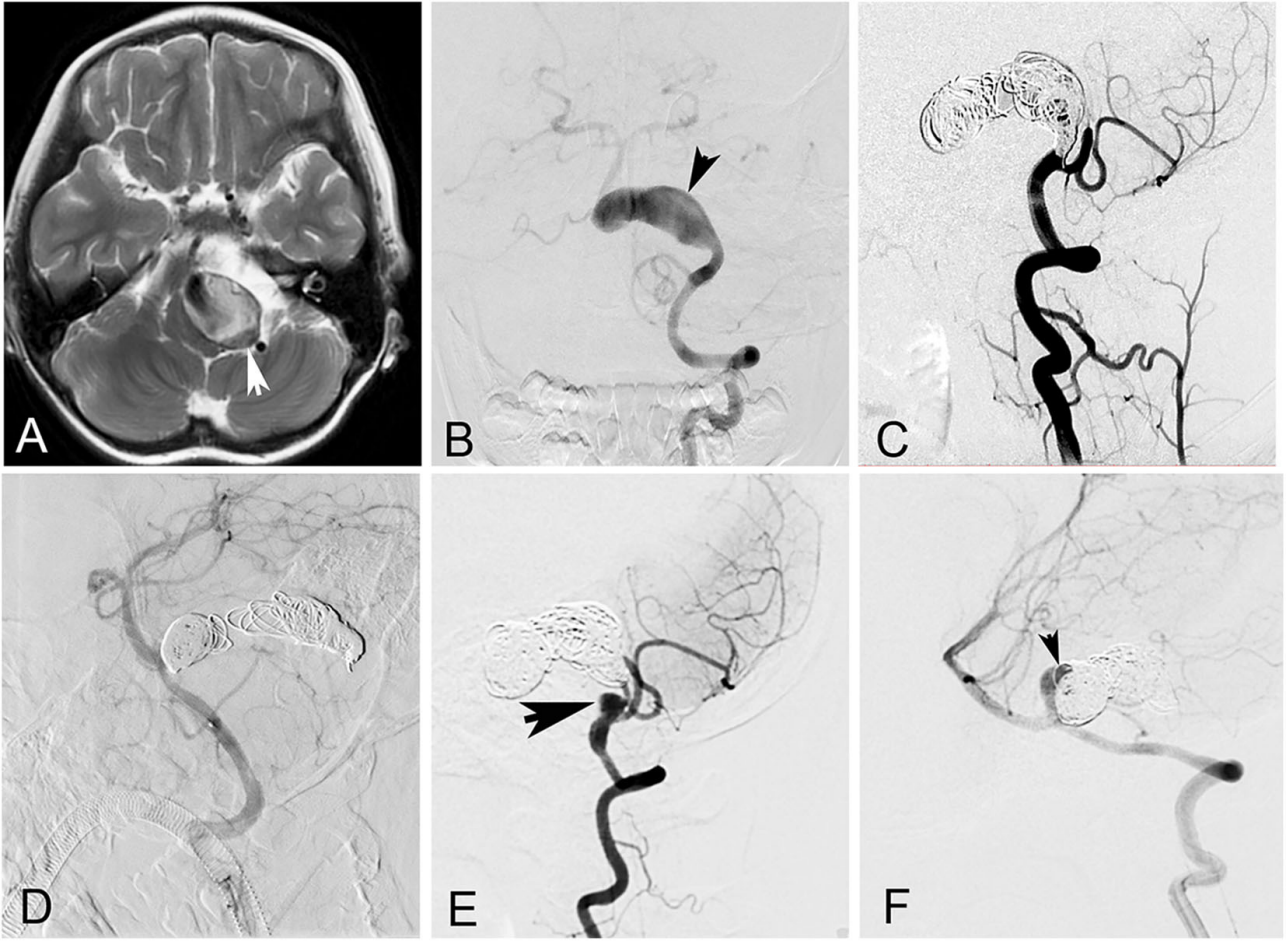
A 6-year-old girl with a giant vertebrobasilar dissecting aneurysm. **(A)** Magnetic resonance imaging showed a giant vertebrobasilar dissecting aneurysm with intramural hematoma. **(B)** Left vertebral anteroposterior angiogram revealed the aneurysm. **(C)** Left vertebral artery and the aneurysm were occluded with coils at lateral angiogram. **(D)** Right vertebral was patency after the procedure at lateral angiogram. **(E,F)** Left and right vertebral lateral angiogram at follow-up showed the aneurysm recanalized (arrows).

**Figure 3:**
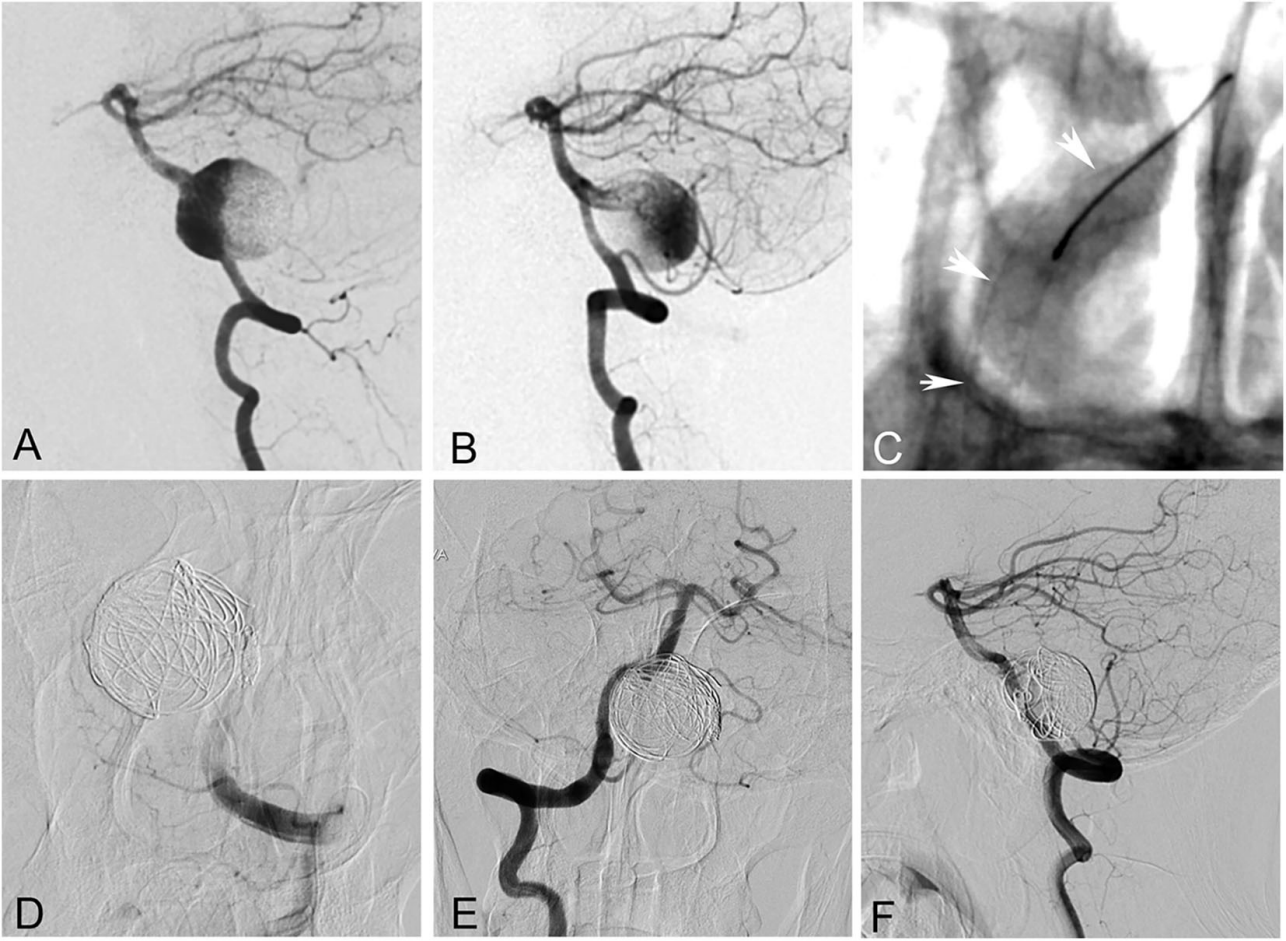
A 55-year-old woman with a giant saccular aneurysm at the vertebrobasilar junction. **(A,B)** Bilateral vertebral angiogram showed the aneurysm. **(C)** The right vertebral and basilar arteries were reconstructed with a pipeline embolization device. **(D)** The left vertebral artery and the aneurysm were occluded with coils. **(E,F)** Right vertebral angiogram showed complete occlusion of the aneurysm after the procedure. However, the patient died of brainstem function failure 3 h later after worsening of mass effect.

The patient cohort comprised 21 men (77.8%) and six women (22.2%). The mean age of the patients was 42.7 ± 18.9 years (ranging from 6 to 65 years). The most common presenting symptom was compressive symptoms, present in 15 patients (55.6%). Nine patients (33.3%) presented with headache, two patients (7.4%) with ischemic stroke, and one (3.7%) with asymptomatic lesion. None of the GVBAs was ruptured. Among the 27 GVBAs, 23 aneurysms were fusiform aneurysm with intramural hematoma (Figure 2) and four aneurysms were saccular type (Figure 3). The average maximal diameter of aneurysms was 29.2 ± 4.4 mm (range, 25.0–39.4 mm). The location of GVBAs was the vertebral artery in 21 cases, basilar artery in three, and vertebral–basilar junction in three.

### Endovascular Treatment Modality and Outcome

Endovascular treatment was technically feasible in all 27 cases. Five dissecting GVBAs received internal trapping. Ten aneurysms underwent stent-assisted coil embolization (single stent, *n* = 1; two stents, *n* = 6; three stents, *n* = 3). Four aneurysms were treated with sole stenting (single stent, *n* = 1; two stents, *n* = 2; three stents, *n* = 1). Eight aneurysms were treated with flow diverters (single flow diverter, *n* = 2; single flow diverter with coils, *n* = 5; four flow diverters, *n* = 1).

None of the patients had intraoperative complications. Among the 27 patients, three patients who underwent flow-diverter deployment suffered from periprocedural complications and subsequent death: one patient died from aneurysmal hemorrhage 4 days with four overlapping flow diverters and the other two died of brainstem failure resulting from compression with single flow diverter adjunctive coils. Clinical follow-up was established for the remaining 24 patients with a mean duration of 14.5 ± 7.1 months (range, 6–32 months); however, angiographic follow-up was provided only for 23 (92.3%) patients (6.9 ± 4.0 months), as one patient died of longer complication (brainstem failure resulting from compression) relatively early in the follow-up period. Of the 24 patients, 20 had a favorable clinical outcome and four with unfavorable clinical outcome during the follow-up period. Eight presented with an unfavorable angiographic outcome, three of whom ultimately died because of severe brainstem compression. Of the eight angiographically unfavorable patients, two were treated initially with internal trapping, three with stent-assisted coils, and three with overlapping stents. Four patients underwent repeat procedures. Despite two patients undergoing repeat internal trapping, the MR image still showed enlargement of the GVBAs at 2-year follow-up. The other two patients were re-treated by stent-assisted coiling and overlapping stents, respectively. We compared and summarized the unfavorable outcomes with different treatments for GVBAs (Table 2).

**Table 2:** Comparison of unfavorable outcomes with different treatments for giant vertebrobasilar aneurysms.

	**Stent-assisted coiling, *n* = 10**	**Sole stenting, *n* = 4**	**Internal trapping, *n* = 5**	**Flow diverter, *n* = 8**
Unfavorable angiographic outcomes	3 (30.0%)	3 (75.0%)	2 (40.0%)	0 (0.0%)
Unfavorable clinical outcomes	3 (30.0%)	0 (0.0%)	1 (20.0%)	3 (37.5%)
Unfavorable angiographic or clinical outcomes	4 (40.0%)	3 (75.0%)	2 (40.0%)	3 (37.5%)

## Discussion

GVBAs are always associated with high morbidity and mortality during the course of natural history if left untreated (11). Although these lesions pose an increased risk to treatment, intervention is usually considered necessary because of the rapidly changing morphology and progressive mass effect (12). Therefore, the primary purpose of this study was to evaluate the safety and efficacy of endovascular treatment for GVBAs. Of the 27 patients with 27 GVBAs included, 12 patients presented with an unfavorable angiographic or clinical outcome. Of these 12 patients, eight GVBAs demonstrated an unfavorable angiographic outcome at follow-up and six patients died of post-operative complications and brainstem compression during the follow-up period. The outcome of treating GVBAs with current endovascular modalities therefore seems questionable and unpredictable. Our results suggest that patients with GVBAs may not derive optimal benefit from endovascular treatment.

At present, appropriate endovascular treatment of GVBAs is very challenging. As summarized in Table 3, there is significant high mortality, permanent morbidity rate of GVBAs with endovascular treatment. Treatment decisions about endovascular modalities are based on evaluation of the location, collateral blood supply, and the important arterial branches if involved (17). All features of GVBAs must be taken into account to undertake the best approach for each patient. If there is a sufficient compensatory blood supply, internal trapping with the occlusion of aneurysm, and parent artery is our first choice. Although previous studies have proved that internal trapping is an effective therapy for this lesion, with a satisfactory long-term outcome (13, 18), it may be not effective in some cases. For instance, GVBAs could continue to enlarge even after deployment of internal trapping. Iihara et al. (19) reported that after a partially thrombosed giant aneurysm of the vertebral artery was treated by internal trapping, the aneurysm continued to enlarge. Formation of intramural hematoma may be a necessary critical event for GVBAs to become progressive (20). The hypothesis for recurrent GVBAs after treatment with internal trapping states that the vasa vasorum in the intramural hematoma is fragile and could cause repetitive intramural hemorrhage, resulting in enlargement of the aneurysm (21). In line with this implication, in our study aneurysm recurrence was observed in four GVBAs that were treated or re-treated by internal trapping.

**Table 3:** Literature review of endovascular treatment for giant vertebrobasilar aneurysms.

**Author, year**	**No of Pts/GVBAs**	**Mean (range) age, years**	**Size(mm) Median (range)**	**Treatment method (%)**	**Perioperative IE (%)**	**Radiologic follow-up (months) median (range)**	**CO at final follow-up (%)**	**Retreatment (%)**	**Good clinical outcome at follow-up (%)**	**Delayed IE (%)**	**Morbidity, % (mortality, %)**
Limaye et al. (1)	22/22	37 (13–63)	>25	Internal trapping (59.0), Flow reversal (22.7), Flow diversion (9.1), Coiling (4.6), Stent-assisted coiling (4.6)	(9.1)	(1–60)	17 (94%)	0 (0)	14 (63.6)	–	22.7 (18.2)
Ge et al. (6)	7/7	43.9 (2–77)	31 (25–40)	Stent-assisted coiling (71.4), Flow diversion (14.3), Coiling (14.3),	–	14.5 (7–22)	1 (14.5)	0 (0)	1 (14.3)	–	0 (85.7)
Lubicz et al. (13)	13/13	47.9 (15–65)	35 (25–60)	Internal trapping (100)	1 (7.7)	25.9 (12–48)	12 (92.3)	1 (7.7)	12 (92.3)	0	0 (7.7)
Siddiqui et al. (14)	2/2	42 (42)	36.4 (35.6–37.1)	Flow diversion (100)	2 (100)	6 (6)	–	–	–	–	0 (100)
Meckel et al. (15)	8/8	55.1 (34–72)	> 25	Flow diversion (100)	1 (12.5)	16.6 (0.5–34)	3 (37.5)	1 (12.5)	4 (50)	1 (12.5)	0 (50)
Toth et al. (16)	3/3	46.6 (30–60)	28.3 (27–29)	Flow diversion (100)	2 (66.7)	13 (9–18)	2 (66.7)	0 (0)	1 (33.3)	0 (0)	33.3 (33.3)

*Pts, patients; GVBAs, giant vertebrobasilar aneurysms; IE, ischemic events; CO, complete occlusion*.

GVBAs treated by conventional stents with or without coiling had a high rate of recanalization, for several possible reasons (22–24). First, total occlusion with dense packing was difficult in the case of dissecting aneurysms because of the complex geometry and irregular shape, without a definitive aneurysm neck and a fragile vessel wall (25). Second, owing to their high porosity, conventional stents have a limited flow-diversion effect (26). Although conventional stents are able to maintain the patency of the parent artery, they cannot completely occlude the persistent blood flow into the aneurysm, which could result in coil compaction. Third, GVBAs were physiologically active and dynamic (27, 28), as rapid change or expansion of GVBAs was observed (12). This may be a reason for the aneurysmal growth and the failure of endovascular treatment. In our study, of the 14 cases treated by conventional stents with or without coil embolization, seven presented with a poor prognosis during the follow-up period.

Flow diverters have emerged as a promising option for GVBAs, and some studies have reported a satisfactory outcome using flow diverters (14, 29). Compared with conventional stents, flow diverters are characterized by more metal coverage, providing a scaffold for neointimal tissue formation and thus completing reconstruction of the parent artery. Moreover, flow diverters could cause stagnation of blood flow and promote thrombosis within the aneurysmal sac by its fluid-diverting effect. However, flow diverters have been associated with the risk of catastrophic complications after endovascular treatment. Siddiqui et al. (15) reported that among seven patients with symptomatic vertebrobasilar aneurysms who underwent endovascular treatment with flow diverters, at the last follow-up evaluation four patients had died (two patients with post-treatment aneurysm rupture and the other two lacking improvement in neurologic status) and one presented with severe disability. Similarly, Meckel et al. (16) reported that four of 10 patients with complex vertebrobasilar junction aneurysms treated with flow diverters died as a result of sequelae of subarachnoid hemorrhage, late flow-diverter thrombosis, progressive mass effect, and delayed intracranial hemorrhage, respectively. In our study, among the seven patients treated with flow diverters, three died of subarachnoid hemorrhage or progressive mass effect while the other four had a good prognosis at follow-up. Therefore, clinicians should be cautious in the decision-making process regarding whether or when a flow diverter should be applied in the posterior circulation.

Treatment of GVBAs is usually necessary because of their unfavorable natural history. However, previous studies observed that mortality and morbidity seem to be higher in symptomatic posterior circulation aneurysms after endovascular treatment (14, 15, 30). Perhaps the best strategy for GVBAs is timely discovery and timely treatment before these lesions become symptomatic and chronically enlarged. Regular physical examination including MRI and MRA for the patients with high risk factor (such as family history of GVBAs), might be an optional method for discovery of these lesions early. Moreover, the assessment of HRMRI in GVBAs patients might be identified the high-risk patients with evidence of progression on imaging (8). However, future studies are necessary to clarify these points and provide optimal treatment for patients with GVBAs and further advancements are necessary to provide optimal treatment for patients with GVBAs.

There are limitations to this study. As it is a retrospective study with a limited number of patients, more data on larger numbers of GVBAs are required. Moreover, different interventional materials were used, and there may be patient selection bias.

## Conclusions

The ideal approach to the treatment of GVBAs remains debatable. Endovascular treatment may not halt the progressive course of GVBAs, and continuous follow-up is required. Newer-generation devices may provide more optimal therapy for the management of these complex lesions.

## Data Availability Statement

The datasets analyzed in this article are not publicly available. Requests to access the datasets should be directed to Miao Li, limiao@jlu.edu.cn.

## Ethics Statement

Ethical review and approval was not required for the study on human participants in accordance with the local legislation and institutional requirements. Written informed consent to participate in this study was provided by the participants' legal guardian/next of kin.

## Author Contributions

ML contributed to the preparation of the manuscript and data collection. HL contributed to data analysis and interpretation. JW contributed to the experimental design and manuscript revision. All authors contributed to the article and approved the submitted version.

## Conflict of Interest

The authors declare that the research was conducted in the absence of any commercial or financial relationships that could be construed as a potential conflict of interest.
